# Long non‐coding RNA DLX6‐AS1 mediates proliferation, invasion and apoptosis of endometrial cancer cells by recruiting p300/E2F1 in DLX6 promoter region

**DOI:** 10.1111/jcmm.15810

**Published:** 2020-09-20

**Authors:** Hui Zhao, Qian Xu

**Affiliations:** ^1^ Department of Gynaecology and Obstetrics Linyi People's Hospital Linyi China

**Keywords:** DLX6, DLX6 antisense RNA 1, E2F1, endometrial cancer, p300, promoter acetylation, RNA‐DNA triplex

## Abstract

Endometrial cancer features abnormal growth of cells of the inner lining of the uterus with the potential to invade to other organs. Accumulating evidence suggests that aberrant expression of long non‐coding RNA (lncRNA) may facilitate cancer progression. The aim of the present study was to identify the molecular mechanisms of the lncRNA known as DLX6 antisense RNA 1 (DLX6‐AS1) in endometrial cancer. Microarray‐based analysis was utilized to predict expression profile and possible function pattern of DLX6‐AS1 and DLX6 in endometrial cancer, and their expression was quantified in 78 clinically obtained endometrial cancer tissues and also in cell lines. We next assessed the effects of DLX6‐AS1 and DLX6 on proliferation, invasion and apoptosis of endometrial cancer cells. A mouse xenograft model was established to confirm DLX6‐AS1 functions and explore its underlying regulatory mechanisms in vivo. DLX6‐AS1 and DLX6 were highly expressed in endometrial cancer tissues and cells, and their silencing weakened the proliferative and invasive abilities of endometrial cancer cells and tumours, while promoting apoptosis. Mechanistic investigations indicated that DLX6‐AS1 formed a triplex structure with DLX6 *via* interaction with p300/E2F1 acetyltransferase. Thus, we find that functional up‐regulation of DLX6‐AS1 can promote endometrial cancer progression via a novel triplex mechanism that may prove to be great clinical significance for future treatments of endometrial cancer.

## 1 INTRODUCTION

Endometrial cancer is a tumour occurring on the surface of the endometrial lining of the uterus. It is the most common cancer among women in developed countries and is also the third greatest cause of cancer‐related death in women worldwide, leading to 76,000 deaths in 320,000 cases recorded worldwide in 2012.^1^ There are multiple risk factors that contribute to the occurrence of endometrial cancer, including dysregulation of hormone secretion, genetic disorders and certain chronic diseases. However, the principle cause of endometrial cancer is exposure of high levels of estrogens, in conjunction with obesity, which occurs in 40% of patients with endometrial cancer.^2^ Around 10% of endometrial cancer cases are caused by hereditary factors in genetic disorders.^3^ However, most endometrial cancer cases proceed with gradual genomic alterations, including an accumulation of somatic mutations and somatic copy number alterations.^1^ These genomic alterations associated with endometrial cancer have been comprehensively identified by genome‐wide association studies (GWAS).^4^ For example, the TP53 mutation is rare in type I endometrial cancer, whereas the PTEN mutation is present in 75%‐85% of cases. In contrast, in type II endometrial cancers, TP53 mutation accounts for over 60% of serous and carcinosarcoma subtypes, while PTEN mutation only accounts for less than 20%.^1^ These two features have been applied as biomarkers for diagnosis and prognosis of endometrial cancer.

There is an increasing appreciation of the role of long non‐coding RNA (lncRNA) in cancer development. LncRNAs are transcripts more than 200 nucleotides that play critical roles in the regulation of gene expression, cell cycle, cell apoptosis and necrosis, which also participate in aspects of cancer procession including tumour cell proliferation, migration and invasion.^5^ The molecular functions of lncRNAs have been classified into four types, namely signals, decoys, scaffolds and guides. The category of lncRNA decoys is understood to operate as a sponge for microRNA (miRNA), and further regulate the expression of miRNA targets.^6^ The guide form of lncRNA forms ribonucleoprotein complex with target proteins and recruits them on genomic *cis* elements to regulate gene expression.^7^

The particular lncRNA investigated in this study of endometrial cancer, namely LncRNA DLX6‐AS1, conforms to this guide role and has the capacity of regulating epigenetically the expression of its corresponding sense gene DLX6 expression. Interestingly, it has been reported that lncRNA DLX6‐AS1 can act as a competing endogenous RNA (ceRNA) to down‐regulate miR‐199a and further promote tumour cell proliferation in cervical cancer.^8^ Therefore, it seems that lncRNA DLX6‐AS1 can participate in diverse aspects of cancer procession of different tumours.

In this work, we found that the coding gene DLX6 and the lncRNA DLX6‐AS1 were both highly expressed in endometrial cancer. Silencing either DLX6 or lncRNA DLX6‐AS1 suppressed proliferation and migration of endometrial cancer cells, and promoted cell apoptosis in vitro and in vivo. We found that lncRNA DLX6‐AS1 regulated DLX6 epigenetically via recruiting P300 to its promotor and showed that DLX6 and lncRNA DLX6‐AS1 are proved as oncogenes presenting new therapeutic targets in endometrial cancer.

## 2 MATERIALS AND METHODS

### 2.1 Ethics statement

The study was approved by the Institutional Review Board of Linyi People's Hospital. All participants provided written informed consent prior to participation. The animal protocols and experiment procedures were conducted in accordance with the International Guide for the Care and Use of Laboratory Animals, following a protocol approved by the institution animal research committee.

### 2.2 Microarray‐based analysis

Analysis of the JASPAR database (http://jaspar.genereg.net/) revealed the existence of binding sites of transcription factor E2F1 on the DLX6‐AS1 promoter and the DLX6 promoter. GEPIA database (http://gepia.cancer‐pku.cn/index.html) showed that DLX6‐AS1 and DLX6 were both highly expressed in endometrial cancer and had a strong positive correlation. Furthermore, DLX6‐AS1 was predicted to have a mainly nuclear localization according to analysis of the lncATLAS website (http://lncatlas.crg.eu/). Meanwhile, Random Forest (RF) and Support Vector Machine (SVM) values between the DLX6‐AS1 sequence and p300 protein sequence were in excess of 0.5, as revealed from the RPIseq data set (http://pridb.gdcb.iastate.edu/RPISeq/), suggesting that DLX6‐AS1 might exert a function in modifying histone acetylation by recruiting p300 protein. Prediction results available from the LongTarget website (http://lncrna.smu.edu.cn) showed that DLX6‐AS1 formed a RNA‐DNA triplex with DLX6 *via* Hoogsteen. The DLX6‐AS1 triplex‐forming oligonucleotide (TFO) sequence was TTTTTTTCTTTCTTTCTTTCTTTTTCTTTCTTTTTTTTTTTTTTTATCAGAATGTTCTGTT and that of the DLX6 triplex target site (TTS) was TCAAAGGGAGGAGGAAGGAGGAGGGATGGGAGAGGAGAGGGGGAGGGGGGAATCTGCTCTC.

### 2.3 Study subjects

A total of 78 female patients (mean age: 56.81 ± 12.47 years, ranging from 35 to 74 years) with endometrial cancer diagnosed in Linyi People's Hospital from June 2016 to June 2017, who had not received any anticancer therapies prior to their operation in our study, with diagnosis according to World Health Organization criteria. Patients were excluded if they also suffered from a serious infection, other solid tumours or diseases of the immune system. Endometrium collected by hysterectomy from patients for other reasons but malignancy was used as controls.

### 2.4 Cell treatment

Human endometrial cancer cell lines (RL‐952, HEC‐1‐B, HEC‐1‐A, HHUA and HEC‐251) and human endometrial cell line PA (all purchased from American Tissue Culture Collection, Rockville, MD, USA) were cultured in minimum Eagle's medium (MEM) (Gibco, Grand Island, NY, USA) containing 10% foetal bovine serum (FBS) (Gibco, Grand Island, NY, USA) and penicillin‐streptomycin in an incubator (Thermo Fisher Scientific Inc, San Jose, CA, USA) at 37ºC with 5% CO_2_. The expression of DLX6‐AS1 in RL‐952, HEC‐1‐B, HEC‐1‐A, HHUA, HEC‐251 and PA cells was determined by reverse transcription‐quantitative polymerase chain reaction (RT‐qPCR). Cell in the logarithmic phase was detached by trypsin and seeded in a 6‐well plate at a density of 1 × 10^5^ cells/well for 24 hours of conventional culture. When cell confluence reached approximately 75%, cell transfection was performed for 48 hours by delivering vector [overexpression (oe)‐negative control (NC) group] or DLX6‐AS1‐vector (oe‐DLX6‐AS1 group; Shanghai GenePharma Co., Ltd., Shanghai, China), dimethyl sulphoxide (DMSO) and C464 (p300/CBP inhibitor) with reference to the manual of Lipofectamine 2000 (Invitrogen Inc, Carlsbad, CA, USA). The transfection efficiency of sh‐DLX6‐AS1 was detected by RT‐qPCR.^9^

### 2.5 RNA isolation and quantitation

Total RNA was extracted using Trizol (15596026, Invitrogen Inc, Carlsbad, CA, USA). RNA was reversely transcribed to cDNA according to instructions in the PrimeScript RT reagent kit (RR047A, Takara, Tokyo, Japan). The synthesized cDNA was subjected to RT‐qPCR using the Fast SYBR Green PCR kit (Applied Biosystems, Oyster Bay, NY, USA) and the ABI PRISM 7300 RT‐qPCR system (Applied Biosystems, Oyster Bay, NY, USA). Triplicates were set for each well. With glyceraldehyde‐3‐phosphate dehydrogenase (GAPDH) as the internal control, the fold changes of DLX6‐AS1 expression were calculated by means of the relative quantification (2^−ΔΔCt^ method).^10^ Primer sequences are shown in Table 1.

**Table 1:** Primer sequences for reverse transcription‐quantitative polymerase chain reaction

Gene	Primer sequences
DLX6‐AS1	F: 5′‐CTGTTTTTGGCCATTGCGGA‐3′
	R: 5′‐ATGTTTGGAGGTTCCCCACC‐3′
DLX6	F: 5′‐CGGAAGCCTCGGACCATTTA‐3′
	R: 5′‐ACTGTGTCTGCTGAAAGCGA‐3′
GAPDH	F: 5′‐GAAGACGGGCGGAGAGAAAC‐3′
	R: 5′‐CCATGGTGTCTGAGCGATGT‐3′

Abbreviations: DLX6‐AS1, DLX6 antisense RNA 1; F, forward; GAPDH, glyceraldehyde‐3‐phosphate dehydrogenase; R, reverse.

### 2.6 Western blot analysis

Cells were harvested and lysed by enhanced radioimmunoprecipitation assay (RIPA) lysis (Boster Biological Technology Co., Ltd., Wuhan, Hubei, China) containing protease inhibitor. Protein concentration was determined with a bicinchoninic acid (BCA) kit (Boster Biological Technology Co., Ltd., Wuhan, Hubei, China). The protein was separated by 10% sodium dodecyl sulphate‐polyacrylamide gel electrophoresis and transferred onto a polyvinylidene fluoride membrane. The membrane was treated with 5% bovine serum albumin (BSA) at room temperature for 2 hours to block non‐specific binding and then incubated at 4ºC overnight with diluted primary rabbit antibody to DLX6 (ab137079, 1:2000, Abcam Inc, Cambridge, MA, USA). After washing with phosphate‐buffered saline Tween‐20 (PBST) three times, the horseradish peroxidase (HRP)‐conjugated secondary goat anti‐rabbit antibody (ab205719, 1:2000, Abcam Inc, Cambridge, MA, USA) was applied for a 1‐h incubation at room temperature, followed by three PBST washes. The membrane was then visualized by enhanced chemiluminescence (Millipore, Temecula, CA, USA). The grey value was quantified and analysed using ImageJ software. GAPDH expression was used as the loading control. Each reaction was run in triplicate.^10^

### 2.7 5‐ethynyl‐2’‐deoxyuridine (EdU) staining

Cells in the logarithmic growth phase were plated in a 24‐well plate, with triplicate wells set for each group. EdU (C10341‐1, Ribo Biotechnology Co., Ltd., Guangzhou, Guangdong, China) was added into the medium to a final concentration of 10 µmol/L, followed by 2‐h incubation. After removal of the medium, cells were fixed in phosphate buffer saline (PBS) containing 4% paraformaldehyde at room temperature for 15 minutes, washed twice with PBS containing 3% BSA and incubated with PBS containing 0.5% Triton‐100 at room temperature for 20 minutes, followed by another two washes in PBS containing 3% BSA. Then, 100 µL Apollo^®^ 567 (Ribo Biotechnology Co., Ltd., Guangzhou, Guangdong, China) was added into each well for 30‐min incubation at room temperature in the dark, followed by another two washes with PBS containing 3% BSA. Then, the sample was stained with 1 × Hoechst 33342 for 30 minutes and washed three times in PBS. The number of EdU positive cells (red) in randomly selected fields was counted under a fluorescence microscope (FM‐600, Shanghai Pudan Optical Instrument Co., Ltd., Shanghai, China). Each experiment was run in triplicate.

### 2.8 Transwell assay

The basement membrane of apical chambers in Transwell chamber was coated with Matrigel (Becton, Dickinson and Company, Franklin L., New Jersey, USA) and allowed to stand at 37ºC for 30 minutes to allow polymerization. The basement membrane was hydrated before use. Cells were cultured in serum‐free medium for 12 hours, collected, and re‐suspended in serum‐free medium (1 × 10^5^ cells/mL). The basolateral chamber was added with medium containing 10% FBS, and 100 μL cell suspension was added into the Transwell chamber for incubation at 37ºC for 24 hours. Cells that did not invade the Matrigel surface was removed gently with cotton swabs. Following fixation in 100% methanol, cells were stained in 1% toluidine blue (Sigma‐Aldrich Chemical Company, St Louis, MO, USA). The number of invading cells was counted under an inverted optical microscope in five randomly selected fields of view. Each experiment was run in triplicate.

### 2.9 Flow cytometry

The endometrial cancer cells were seeded in 6‐well plates containing penicillin‐streptomycin‐free medium at a density of 1 × 10^6^ cells/well. At 48 hours post‐transfection, cells were collected, washed twice and stained by fluorescein isothiocyanate (FITC)‐Annexin V and propidium iodide (PI) (Becton, Dickinson and Company, Franklin L., New Jersey, USA) following the steps described in the instructions of the Annexin V‐FITC/PI apoptosis detection kit (Becton, Dickinson and Company, Franklin L., New Jersey, USA). Cell apoptosis was analysed using Fluorescence Activated Cell Sorting (Becton, Dickinson and Company, Franklin L., New Jersey, USA). Each experiment was run in triplicate.^11^

### 2.10 Fluorescence in situ hybridization (FISH)

The subcellular localization of DLX6‐AS1 was detected by FISH assay. Following the manual of the RiboTM lncRNA FISH Probe Mix (Red) kit (Ribo Biotechnology Co., Ltd., Guangzhou, Guangdong, China), DLX6‐AS1 probes were custom‐made based on the DLX6‐AS1 sequences. Coverslips were placed in the 6‐well plates where the cells were seeded. When cell confluence had reached about 80% on the next day, the coverslip was washed with PBS, fixed with 1 mL of 4% paraformaldehyde and treated with proteinase K (2 μg/mL), glycine and acetylation reagent. Then, cells were prehybridized with 250 μL pre‐hybridization solution at 42ºC for 1 h. After the removal of pre‐hybridization solution, 250 μL hybridization solution (300 ng/mL) containing DLX6‐AS1 probes was added for hybridization at 42ºC overnight. After three PBST washes, the nucleus was stained with PBST‐diluted 4',6‐diamidino‐2‐phenylindole (DAPI; 1:800) in a 24‐well plate for 5 minutes, followed by three PBST washes. Five fields of view were randomly selected for microscopic observation and photography under a fluorescence microscope (Olympus Corp., Tokyo, Japan). Each experiment was run in triplicate.^12^

### 2.11 Subcellular fraction

Nucleus and cytoplasm were separated following the manufacturer's instructions in the PARIS kit (Life Technologies, Carlsbad, CA, USA).^13^

### 2.12 RNA immunoprecipitation (RIP)

The binding between DLX6‐AS1 and p300 protein was detected by the RIP kit (Millipore, Temecula, CA, USA). Cells were washed with pre‐cold PBS, and the supernatant was discarded. RIPA lysis (P0013B, Beyotime Biotechnology Co., Ltd., Shanghai, China) of equal volume was added to lyse cells in the ice‐bath for 5 minutes. The supernatant was obtained through centrifugation (7043.4 *g*, 4ºC, 10 minutes). A portion of the cell extract was used as Input and the other part was used for coprecipitation with antibody. Magnetic beads (50 μL) were washed, re‐suspended in 100 μL RIP Wash Buffer and incubated with the corresponding antibodies (5 μg), including rabbit antibody to p300 (1:100, ab10485, Abcam Inc, Cambridge, MA, USA) for a 30‐minutes incubation at room temperature, where rabbit anti‐human antibody to immunoglobulin G (IgG) (1:100, ab109489, Abcam Inc, Cambridge, MA, USA) served as NC. The magnetic bead‐antibody complex was washed, re‐suspended in 900 μL RIP Wash Buffer and incubated with 100 μL cell extract at 4ºC overnight. The sample was washed three times and placed on the magnetic base for collection of the magnetic bead‐protein complex. Following detachment by protease K, RNA was extracted from each sample and its Input for further DLX6‐AS1 quantification by RT‐qPCR.^14^

### 2.13 RNA pull‐down assay

Endometrial cancer cells were transfected with wild‐type (WT) biotinylated DLX6‐AS1 (50 nmol/L) and mutant type (MUT) biotinylated DLX6‐AS1 (50 nmol/L), respectively, for 48 hours, and washed with PBS, followed by incubation with specific cell lysis buffer (Ambion, Austin, Texas, USA) for 10 minutes. Then, the cell lysate was sub‐packed (50 µL). The remaining lysate and M‐280 streptavidin magnetic beads (Sigma‐Aldrich Chemical Company, St. Louis, MO, USA) pre‐coated by RNase‐free yeast tRNA (Sigma‐Aldrich Chemical Company) were incubated at 4ºC for 3 hours. The sample was washed twice in cold lysis buffer, three times in low‐salt buffer solution, and once in high‐salt buffer solution. Total protein was extracted with high‐efficiency RIPA lysis buffer, and the p300 expression was determined by Western blot analysis.^15^, ^16^

### 2.14 Chromatin IP (ChIP) assay

Following treatment with 4% formaldehyde (final concentration: 1%), cells were subjected to ultrasonication and incubated with rabbit antibody against E2F1 (ab179445, 1:50, Abcam Inc, Cambridge, MA, USA) for binding to E2F1‐DLX6‐AS1 and the E2F1‐DLX6 promoter. Next, Protein A Agarose/SaLmon Sperm DNA was added to form complexes of E2F1 antibody‐E2F1‐DLX6‐AS1 and E2F1 antibody‐E2F1‐DLX6 promoter. The precipitate was washed to remove non‐specific binding. The complex enriched with E2F1‐DLX6‐AS1 and E2F1‐DLX6 promoter was then obtained and de‐crosslinked. The purified fragments enriched with DLX6‐AS1 and DLX6 promoter were detected by PCR with Input as the internal reference. Each experiment was run in triplicate.^17^

### 2.15 In vitro triplex pull‐down assay

Primers were designed to amplify the 5’untranslated region (5’UTR) fragment (TSS) from the genome, and PCR products were digested by exonuclease I. Then, 1 pmoL biotin‐labelled DLX6‐AS1 TFO probe was added and incubated in triplex hybridization solution at 37°C for 30 minutes. At the same time, the Control oligo NC group (without DLX6‐AS1 TFO sequences) and the blank control group (without RNA) were set. The RNA‐DNA complex was incubated with streptavidin magnetic beads at 37°C for 40 minutes and washed once with buffer solution. DNA that bound to RNA was removed by washing with 1% sodium dodecyl sulphate, 50 mmol/L Tris‐HCl (pH = 8) and 10 mmol/L ethylenediaminetetraacetic acid (EDTA) at 65°C for five min, followed by treatment with RNase A (50 ng/mL, 37°C, 30 minutes) and protease K (200 ng/mL, 15°C, 15 minutes). After extraction by phenol‐chloroform and purification with ethanol, DNA was analysed by RT‐qPCR, with results normalized as described above.^18^

### 2.16 Electrophoretic mobility shift assay (EMSA)

T4 polynucleotide kinase (T4‐PNK) was used to label the end of DLX6 TSS fragment ([γ‐^32^P] ATP 5000 Ci/mmol, 10 mCi/mL). The labelling system (10 μL) consisted of 1 μL [γ‐^32^P] ATP, 1 μL T4‐PNK, 1 μL 10 × T4‐PNK buffer, 300‐500 ng DLX6 DNA and sterile deionized water. Following reaction at 37°C for ten min, 1 μL 0.5 mol/ L EDTA was added for termination. The labelled DNA was reacted with synthesized DLX6‐AS1 TFO in 10 μL binding system [1 mmol/L MgCl_2_, 0.5 mmol/L EDTA, 0.5 mmol/L DTT, 50 mmol/L NaCl, 10 m Tris‐HCI (pH = 7.5) and 0.05 mg/mL poly‐(dl‐dC)] at room temperature for 20 minutes. The sample was then subjected to 4% non‐denaturing polyacrylamide gel electrophoresis and exposed to radiation at 60°C for visualization of retardant belts. Each experiment was run in triplicate.

### 2.17 In vivo triplex capture assay

Nuclei (2 × 10^6^) were isolated from cells that had been transfected with DLX6 TSS fragments and then incubated with 8 pmoL biotinylated DLX6‐AS1 TFO probes in triplex buffer solution [10 mmol/L Tris‐HCl (pH = 7.5), 20 mmol/L KCl, 10 mmol/L MgCl_2_ and 100 U RNAsin] for 1 hour. Meanwhile, the Control oligo NC group (without DLX6‐AS1 TFO sequences) and the blank control group (without RNA) were designed. The excess RNA was removed by centrifugation in a 0.88 mol/L sucrose gradient. After ultrasonication and centrifugation (5031 *g*, 5 minutes) isolated nuclei were incubated with streptavidin magnetic beads for 40 minutes. DNA that bound to RNA was removed using RNase A and protease K, followed by RT‐qPCR and normalization.^19^

### 2.18 Xenograft tumour in nude mice

A total of 32 healthy nude mice (aged 6‐8 weeks, Beijing Pharmacology Institute of Chinese Academy of Medical Sciences, Beijing, China) were separately housed in a specific pathogen free animal laboratory at constant temperature of 22‐25ºC with constant humidity of 60%‐65% and free access to food and water under a 12‐h light/12‐h dark cycle. Experiments were initiated after one‐week acclimation, with monitoring of health status. Endometrial cancer cells (RL‐952 and HHUA cells) were infected with lentivirus (2 × 10^8^ plaque‐forming unit/mL) of short hairpin RNA (sh)‐NC, sh‐DLX6‐AS1, sh‐DLX6‐AS1 + sh‐DLX6 and sh‐DLX6‐AS1 + oe‐DLX6. The cells with stable transfection were dispersed into a cell suspension (5 × 10^7^ cells/mL). Then, for tumorigenesis, 0.2 mL cell suspension was subcutaneously inoculated into the left armpit of BALB/c nude mice (n = 8/each group) using a 1 mL syringe. After 5 weeks, mice were euthanized with the size and weight of tumour recorded. The DLX6 expression was determined. Each experiment was run in triplicate.

### 2.19 Statistical analysis

SPSS 21.0 software (IBM Corp., Armonk, NY, USA) was applied for data analysis. The measurement data were expressed as mean ± standard deviation. Comparison of paired data between two groups following normal distribution and homogeneity of variance was conducted by paired t test while unpaired data were conducted by unpaired t test. Comparison among multiple groups was conducted by one‐way analysis of variance, followed by Tukey's post hoc test. Comparison at different time points within group was conducted by repeated measures analysis of variance, followed by Bonferroni post hoc test. Values of *P* < 0.05 were considered statistically significant.

## 3 RESULTS

### 3.1 DLX6‐AS1 is highly expressed in endometrial cancer

**Figure 1:**
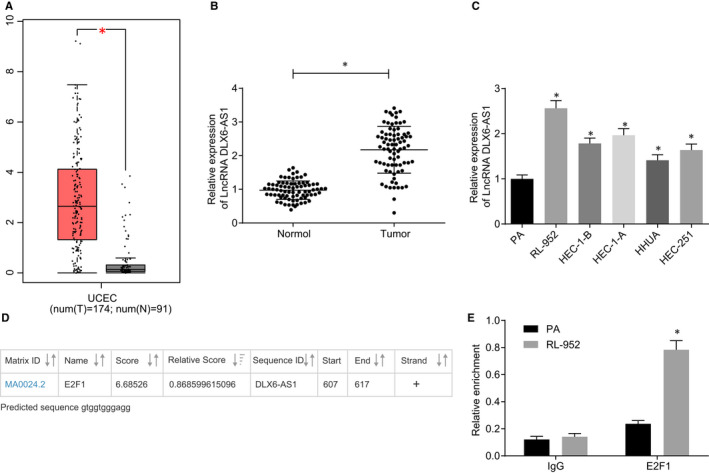
High expression of DLX6‐AS1 is found in endometrial cancer. A, Expression profile of DLX6‐AS1 analysed in GEPIA database. B, Relative expression of DLX6‐AS1 in endometrial cancer tissues and normal tissues determined by RT‐qPCR. C, Relative expression of DLX6‐AS1 in endometrial cancer cell lines determined by RT‐qPCR. D, Binding of E2F1 on the DLX6‐AS1 promoter region predicted on ASPAR website. E, Binding of E2F1 on the DLX6‐AS1 promoter region detected by ChIP assay. *P < 0.05 vs normal tissues or PA cells. The results were measurement data and expressed as mean ± standard deviation. In panel B, unpaired t test was used for comparison (n = 78). In panel C and E, one‐way analysis of variance with Tukey's post hoc test was performed for multiple comparisons. Cell experiments were repeated in triplicate independently

Initially, analysis from GEPIA database (http://gepia.cancer‐pku.cn/index.html) showed that DLX6‐AS1 was highly expressed in endometrial cancer (Figure 1A), which was further confirmed by RT‐qPCR of the patient samples (Figure 1B). The correlation analysis between DLX6‐AS1 and clinicopathological features of patients with endometrial cancer is depicted in Table 2, which revealed no significant correlation between DLX6‐AS1 and age or degree of lymphatic metastasis (*P* > 0.05). However, there was a significant correlation of DLX6‐AS1 with histological classification and tumour node metastasis (TNM) stage (*P* < 0.05). Subsequently, RT‐qPCR was performed to determine the DLX6‐AS1 expression in endometrial cancer cell lines, results of which revealed significantly higher DLX6‐AS1 expression in endometrial cancer cell lines (RL‐952, HEC‐1‐B, HEC‐1‐A, HHUA and HEC‐251) when compared with endometrial cells (PA), of which RL‐952 cell line exhibited the highest DLX6‐AS1 expression and HHUA cell line had the lowest expression (*P* < 0.05; Figure 1C). Therefore, the RL‐952 cell line was selected for further experiments. The JASPAR website (http://jaspar.genereg.net/) was employed for binding site prediction (gtggtgggagg) between transcription factor E2F1 and the DLX6‐AS1 promoter, as shown in Figure 1D. ChIP assay for binding between E2F1 and DLX6‐AS1 promoter region showed that enrichment of E2F1 in DLX6‐AS1 promoter region was higher in the RL‐952 cell line than in PA cells (*P* < 0.05; Figure 1E). The aforementioned results indicate that transcription factor E2F1 is enriched in DLX6‐AS1 promoter region, facilitating the DLX6‐AS1 expression in endometrial cancer tissue and cells in association with histological classification and TNM stage.

**Table 2:** DLX6‐AS1 expression levels are associated with clinicopathological features of patients with endometrial cancer

Clinicopathological factor	n	DLX6‐AS1 expression	
		Mean ± SD	*P*‐value
Gender			
Female	78		
Age (years)			
≥60	49	2.14 ± 0.75	0.556
<60	29	2.24 ± 0.67	
Differentiation			
Well	24	1.75 ± 0.70	0.001*
Moderate	23	2.51 ± 0.58	
Poor	31	2.26 ± 0.61	
Lymph node metastasis			
Positive	31	2.26 ± 0.61	0.389
Negative	47	2.12 ± 0.75	
TNM stage			
I	25	1.85 ± 0.79	0.011*
II	22	2.42 ± 0.57	
III	31	2.26 ± 0.61	

Abbreviations: DLX6‐AS1, DLX6 antisense RNA 1; TNM, Tumour Node Metastasis.

* *P* < 0.05 indicated statistical significance.

### 3.2 Silencing DLX6‐AS1 represses endometrial cancer cell proliferation and invasion and contributes to apoptosis

**Figure 2:**
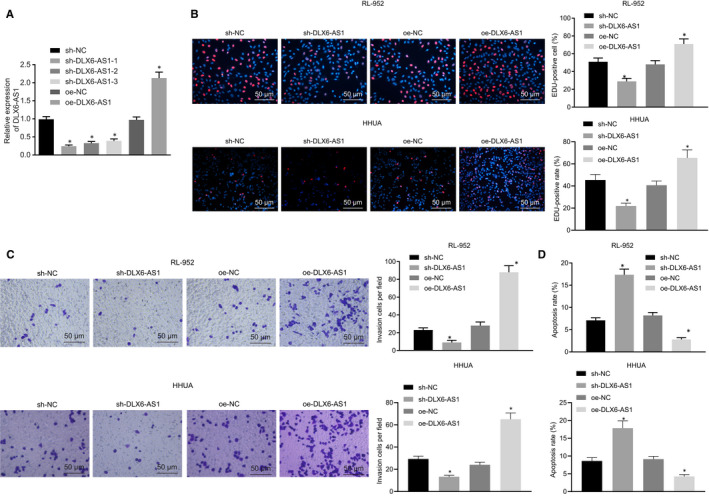
Down‐regulated DLX6‐AS1 inhibits proliferation and apoptosis of endometrial cancer cells while inducing apoptosis. A, Silencing efficiency in RL‐952 and HHUA cell detected by RT‐qPCR. B, RL‐952 and HHUA cell proliferation detected by EdU assay (200 ×). C, RL‐952 and HHUA cell invasion detected by Transwell assay (200 ×). D, RL‐952 and HHUA cell apoptosis detected by flow cytometry. *P < 0.05 vs the sh‐NC group (RL‐952 and HHUA cells treated with sh‐NC) or the oe‐NC group (RL‐952 and HHUA cells treated with oe‐NC). The results were measurement data and expressed as mean ± standard deviation. Comparison among multiples groups was conducted by one‐way analysis of variance, followed by Tukey's post hoc test. Cell experiments were repeated three times independently

We next focus on the potential role of DLX6‐AS1 in regulating biological behaviours of endometrial cancer cells. After DLX6‐AS1 was silenced or overexpressed in RL‐952 and HHUA cells, RT‐qPCR was conducted to detect the silence efficiency of DLX6‐AS1 (Figure 2A), results of which showed that sh‐DLX6‐AS1‐1, sh‐DLX6‐AS1‐2 and sh‐DLX6‐AS1‐3 exhibited lower expression when compared with sh‐NC. Among these, sh‐DLX6‐AS1‐1 caused the lowest expression (*P* < 0.05). Besides, DLX6‐AS1 expression was increased by treatment with oe‐DLX6‐AS1 compared with oe‐NC treatment. Therefore, the sh‐DLX6‐AS1‐1 sequence was selected for subsequent experiments. Then, effects of DLX6‐AS1 on RL‐952 and HHUA cell proliferation, invasion and apoptosis were explored by means of EdU assay (Figure 2B), Transwell assay (Figure 2C) and flow cytometry (Figure 2D), respectively. Results revealed that RL‐952 and HHUA cells treated with sh‐DLX6‐AS1 presented reduced proliferation, suppressed invasion, and enhanced apoptosis, while oe‐DLX6‐AS1 treatment induced opposite effects in RL‐952 and HHUA cells (*P* < 0.05). The evidence suggests that silencing of DLX6‐AS1 impedes proliferation and invasion of endometrial cancer cells while promoting apoptosis, while such alternations can be rescued by restoration of DLX6‐AS1.

### 3.3 DLX6 is highly expressed in endometrial cancer

**Figure 3:**
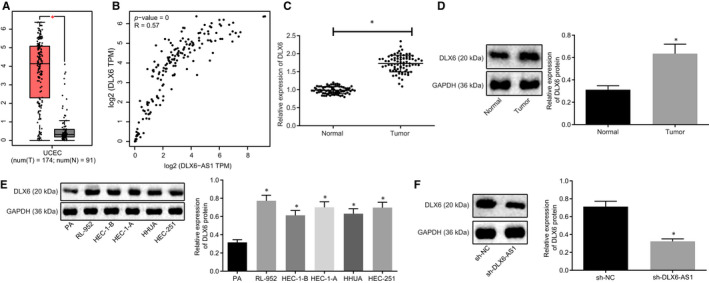
High expression of DLX6 is found in endometrial cancer. A, Expression profile of DLX6 analysed in GEPIA database. B, Correlation between DLX6‐AS1 and DLX6 analysed in GEPIA database. C, Relative expression of DLX6 in endometrial cancer tissues and normal tissues determined by RT‐qPCR. D, Relative expression of DLX6 in endometrial cancer tissues and normal tissues determined by Western blot analysis. E, Relative expression of DLX6 in endometrial cancer cell lines determined by Western blot analysis. F, Relative expression of DLX6 in endometrial cancer cells determined by Western blot analysis after silencing DLX6‐AS1. *P < 0.05 vs normal tissues or PA cells or sh‐NC treatment. The results were measurement data and expressed as mean ± standard deviation. Comparison between two groups was conducted by non‐paired t test. Comparison among multiples groups was conducted by one‐way analysis of variance, followed by Tukey's post hoc test. Cell experiment was repeated three times independently

According to GEPIA database (http://gepia.cancer‐pku.cn/index.html), DLX6 is also highly expressed in endometrial cancer and positively correlated to DLX6‐AS1 (Figure 3A‐B). RT‐qPCR and Western blot analysis for DLX6 expression revealed higher DLX6 expression in endometrial cancer tissues than in normal tissues (*P* < 0.05; Figure 3C‐D). Western blot analysis also showed that endometrial cancer cell lines (RL‐952, HEC‐1‐B, HEC‐1‐A, HHUA and HEC‐251) had significantly higher DLX6 expression than did PA cells (*P* < 0.05; Figure 3E). Western blot analysis after silencing DLX6‐AS1 revealed significantly decreased DLX6 expression when compared with sh‐NC treatment (*P* < 0.05; Figure 3F). Taken together, these findings suggest high expression of DLX6 in endometrial cancer tissue and cells.

### 3.4 Silencing DLX6 weakens endometrial cancer cell proliferation and invasion while inducing apoptosis

**Figure 4:**
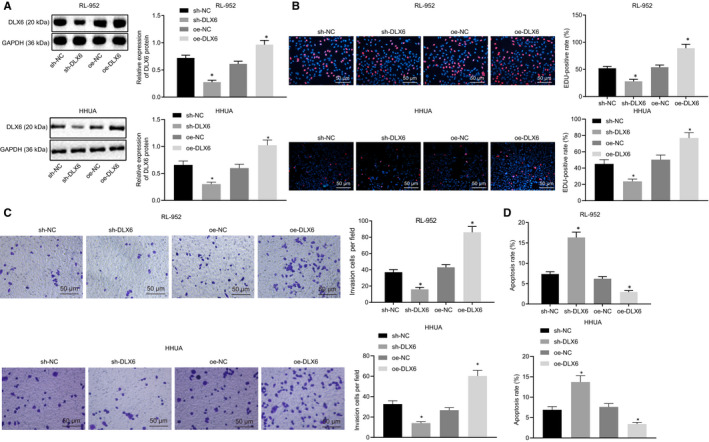
Down‐regulated DLX6 inhibits proliferation and apoptosis of endometrial cancer cells while inducing apoptosis. A, Silence efficiency in RL‐952 and HHUA cells detected by Western blot analysis. B, RL‐952 and HHUA cell proliferation detected by EdU assay (200 ×). C, RL‐952 and HHUA cell invasion detected by Transwell assay (200 ×). D, RL‐952 and HHUA cell apoptosis detected by flow cytometry. *P < 0.05 vs the sh‐NC group (RL‐952 and HHUA cells treated with sh‐NC) or the oe‐NC group (RL‐952 and HHUA cells treated with oe‐NC). The results were measurement data and expressed as mean ± standard deviation. Comparison among multiple groups was conducted by one‐way analysis of variance (ANOVA), followed by Tukey's post hoc test. Cell experiments were repeated three times independently

After confirming the aberrantly expressed DLX6 in endometrial cancer, we explored its effects on biological functions of endometrial cancer cells. Western blot analysis showed that sh‐DLX6 led to significantly lower DLX6 expression in RL‐952 and HHUA cells, while oe‐DLX6 treatment led to significantly higher DLX6 expression (*P* < 0.05; Figure 4A). Then, EdU assay (Figure 4B), Transwell assay (Figure 4C) and flow cytometry (Figure 4D) were conducted to detect RL‐952 and HHUA cell proliferation, invasion and apoptosis, respectively. Results demonstrated that RL‐952 and HHUA cells treated with sh‐DLX6 exhibited attenuated proliferation and invasive ability and augmented apoptosis, while oe‐DLX6 delivery induced the opposite effect (*P* < 0.05). Thus, silencing DLX6 exerts inhibitory effects on endometrial cancer cell proliferation and invasion and promoting effects on apoptosis.

### 3.5 DLX6‐AS1 increases DLX6 expression by recruiting p300/E2F1 to DLX6 promoter

**Figure 5:**
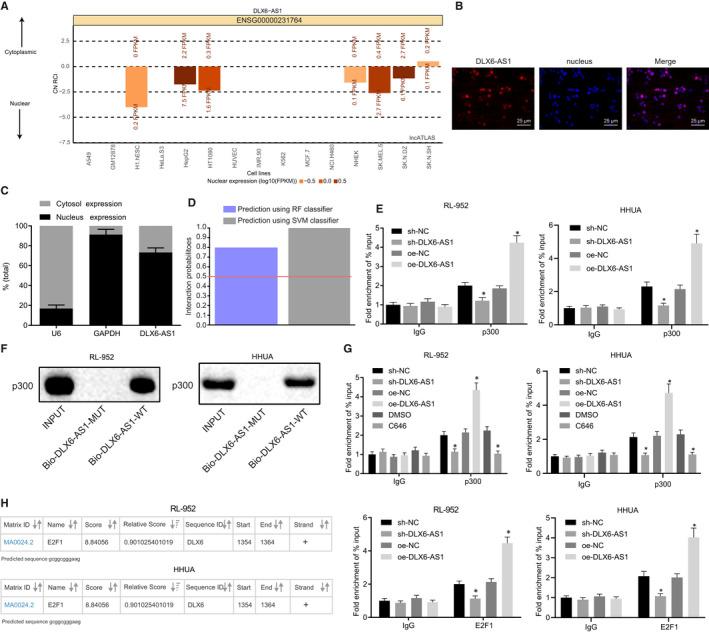
DLX6‐AS1 up‐regulates DLX6 expression by recruiting p300/E2F1 to the DLX6 promoter. A, subcellular localization of DLX6‐AS1 predicted on lncATLAS. B, Subcellular localization of DLX6‐AS1 detected by FISH assay (400 ×). C, subcellular localization of DLX6‐AS1 following subcellular fraction. D, Binding ability between sequences of DLX6‐AS1 and p300 predicted by the RPIseq database. E, Binding between DLX6‐AS1 and p300 detected by RIP. F, DLX6‐AS1 pulling down of p300 detected by RNA pull‐down assay. G, Enrichment of p300 in the DLX6 promoter region detected by ChIP. H, Enrichment of E2F1 in the DLX6 promoter region detected by ChIP. *P < 0.05 vs sh‐NC treatment or oe‐NC treatment or DMSO treatment. The results were measurement data and expressed as mean ± standard deviation. Comparison between two groups was conducted by non‐paired t test. Comparison among multiples groups was conducted by one‐way analysis of variance (ANOVA), followed by Tukey's post hoc test. Cell experiments were repeated three times independently

Subsequently, we explored the regulatory mechanism underlying DLX6‐AS1 and DLX6 effects. The analysis available on the lncATLAS website (http://lncatlas.crg.eu/) revealed that DLX6‐AS1 was mainly localized in the nucleus (Figure 5A), which was in line with results of our FISH assay (Figure 5B) and subcellular fraction study (Figure 5C). The RF and SVM values between DLX6‐AS1 sequence and p300 protein sequences were > 0.5, as revealed from RPIseq (http://pridb.gdcb.iastate.edu/RPISeq/), suggesting that DLX6‐AS1 might modify histone acetylation by recruiting p300 protein (Figure 5D). The binding between DLX6‐AS1 and p300 were detected by RIP assay, which demonstrated that more p300 tended to bind to oe‐DLX6‐AS1 but less p300 bound to sh‐DLX6‐AS1 (*P* < 0.05; Figure 5E). RNA pull‐down assay showed that Bio‐DLX6‐AS1‐MUT was incapable of pulling down p300, whereas Bio‐DLX6‐AS1‐WT was able to pull‐down p300 (Figure 5F). The enrichment of p300 in the DLX6 promoter region was detected by ChIP, which revealed decreased p300 enrichment with sh‐DLX6‐AS1 treatment, but increased enrichment with oe‐DLX6‐AS1 (*P* < 0.05; Figure 5G). Besides, compared with DMSO vehicle treatment, treatment with the p300/CBP inhibitor C464 significantly lowered p300 enrichment in the DLX6 promoter region (*P* < 0.05; Figure 5G). As shown in Figure 1E, transcription factor E2F1 enrichment in DLX6‐AS1 promoter region was elevated. To explore the relation between E2F1 and DLX6, we turned to the JASPAR website (http://jaspar.genereg.net/) for prediction on the binding sequences between E2F1 and DLX6 promoter, which indicated gcggcgggaag. ChIP assay for binding of E2F1 in the DLX6 promoter region showed that sh‐DLX6‐AS1 treatment resulted in less E2F1 enrichment while oe‐DLX6‐AS1 increased E2F1 enrichment (*P* < 0.05; Figure 5H). These findings highlight that DLX6‐AS1 recruits p300/E2F1 to the DLX6 promoter to activate DLX6 expression.

### 3.6 DLX6‐AS1 binds to DLX6 promoter in the form of DNA‐RNA triplex

**Figure 6:**
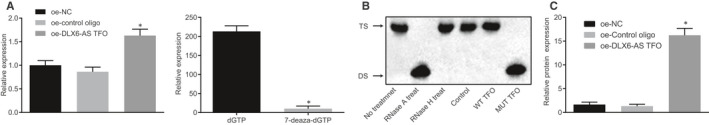
DLX6‐AS1 and DLX6 promoter initiates the DNA‐RNA triplex formation. A, Binding between DLX6‐AS1 and DLX6 promoter detected by in vitro triplex pull‐down assay. B, DNA‐RNA triplex formation between DLX6‐AS1 and DLX6 detected by electrophoretic mobility shift assay. C, Binding between DLX6‐AS1 and DLX6 promoter detected by in vivo triplex capture assay. *P < 0.05 vs the oe‐NC, oe‐control oligo or dGTP group. The results were measurement data and expressed as mean ± standard deviation. Comparison between two groups was conducted by non‐paired t test. Comparison among multiples groups was conducted by one‐way analysis of variance (ANOVA), followed by Tukey's post hoc test. Cell experiments were repeated three times independently

According to predictions in the LongTarget website (http://lncrna.smu.edu.cn), DLX6‐AS1 and DLX6 might form the RNA‐DNA triplex *via* Hoogsteen. The DLX6‐AS TFO sequence was TTTTTTTCTTTCTTTCTTTCTTTTTCTTTCTTTTTTTTTTTTTTTATCAGAATGTTCTGTT and the DLX6 TTS sequence was TCAAAGGGAGGAGGAAGGAGGAGGGATGGGAGAGGAGAGGGGGAGGGGGGAATCTGCTCTC. We then used TFO to detect the ability of DLX6‐AS1 to form the predicted triplex. In vitro triplex, pull‐down assay showed that DLX6 obtained from streptomycin‐coated magnetic beads increased with oe‐DLX6‐AS TFO treatment than with oe‐control oligo treatment. Besides, in studies using 7‐deaza‐dGTP to amplify the DLX6 double‐strand and maturation of TTS, there was decreased DLX6‐AS enrichment, which lowered the binding affinity of DNA double‐strand to DLX6‐AS1 (*P* < 0.05; Figure 6A). The EMSA assay demonstrated that triplex formation was sensitive to RNase A but resistant to RNase H. Next, DLX6‐AS TFO WT and MUT was incubated with ^32^P‐labelled WT DLX6 TTS, respectively, results of which showed that enrichment of MUT DLX6‐AS1 reduced significantly (Figure 6B). To further verify the in vivo binding between DLX6‐AS1 and DLX6, the in vivo triplex capture assay was performed using biotin‐labelled DLX6‐AS1 TFO and endometrial cancer cell lysate. This test revealed significant enrichment of DLX6 on DLX6‐AS1 TFO rather than the oligonucleotide sequence (*P* < 0.05; Figure 6C). To conclude, these findings demonstrate that DLX6‐AS1 binds to the DLX6 promoter in the form of a DNA‐RNA triplex.

### 3.7 Silencing DLX6‐AS1 or DLX6 suppresses endometrial cancer cell growth in vivo

**Figure 7:**
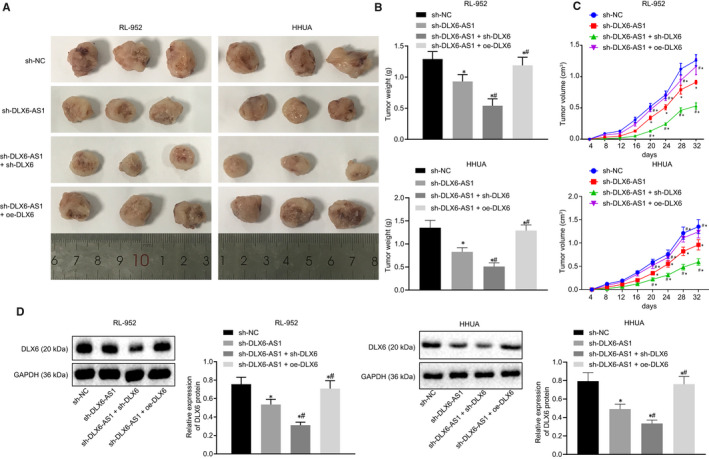
Down‐regulated DLX6‐AS1 and DLX6 exert inhibitory effects on endometrial cancer cell growth in vivo. A, Representative pictures of tumours resected from nude mice. B, Tumour weight of nude mice. C, Tumour growth curve of nude mice. D, Expression of DLX6 determined by Western blot analysis. *P < 0.05 vs the sh‐NC group (nude mice bearing RL‐952 and HHUA cells treated with sh‐NC). ^#^ *P* < 0.05 vs the sh‐DLX6‐AS1 group (nude mice bearing RL‐952 and HHUA cells treated with sh‐DLX6‐AS1). The results were measurement data and expressed as mean ± standard deviation. Comparison among multiples groups was conducted by one‐way analysis of variance (ANOVA), followed by Tukey's post hoc test. Comparison at different time points was analysed by repeated measures analysis of variance, followed by Bonferroni *post hoc* testing. n = 8

Finally, to investigate the effects of DLX6‐AS1 on endometrial cancer cell growth in vivo, we inoculated the transfected RL‐952 and HHUA cells into nude mice, and monitored their tumour weight and growth curves, followed by Western blot analysis for determining the DLX6 expression. Results showed that inoculation of RL‐952 and HHUA cells containing sh‐DLX6‐AS1, sh‐DLX6‐AS1 + sh‐DLX6 and sh‐DLX6‐AS1 + oe‐DLX6 led to smaller tumour size and weight (Figure 7A‐C) along with lower DLX6 expression (Figure 7D) in comparison with sh‐NC (*P* < 0.05). Increased tumour size and weight and DLX6 expression were observed after sh‐DLX6‐AS1 + oe‐DLX6 treatment as compared with sh‐DLX6‐AS1 treatment, whereas there were opposite trends after sh‐DLX6‐AS1 + sh‐DLX6 treatment (*P* < 0.05). To sum up, endometrial cancer cell growth in vivo can be suppressed by silencing DLX6‐AS1 or DLX6.

## 4 DISCUSSION

Endometrial cancer is one of the most common gynaecological cancers all over the world. In low‐grade stages, prognosis of patients with endometrial cancer is optimistic, with an overall 85% survival at five years. However, in high‐grade stages, the prognosis is distinctly poorer, with overall five‐year survival of only 55%.^1^ Hence, it is an urgent matter to identify accurate and efficient biomarkers or targets for prognostic prediction and improved therapeutics of endometrial cancer. Although many coding genes such as TP53 and PTEN have been identified as prognostic biomarkers, lncRNAs have been recently uncovered to have a strong association with the development of endometrial cancer.^20^ Therefore, in this study, we scrutinized the regulation between lncRNA DLX6‐AS1 and the DLX6 coding, with the result that lncRNA DLX6‐AS1 was proved to recruit p300 and E2F1 to form an RNP complex at the promoter region of DLX6. The RNP complex altered the epigenetic status of the promoter and enhanced the transcription level of DLX6. Finally, silencing of either lncRNA DLX6‐AS1 or DLX6 suppressed tumorigenesis of endometrial cancer in vitro and in vivo.

First, we found that both lncRNA DLX6‐AS1 and DLX6 were enriched in endometrial cancer tissues or cell lines, and that silencing lncRNA DLX6‐AS1 or DLX6 inhibited tumour growth and promoted apoptosis. Little is known about lncRNA DLX6‐AS1 in endometrial cancer, but several investigations have shown that lncRNA DLX6‐AS1 is overexpressed in lung adenocarcinoma, renal cell carcinoma, hepatocellular carcinoma, pancreatic cancer and many other kinds of tumours.^10^, ^21^, ^22^ For example, in renal cell carcinoma, lncRNA DLX6‐AS1 could promote cell growth and cancer development via miR‐26a‐mediated up‐regulation of PTEN.^21^ In hepatocellular carcinoma, lncRNA DLX6‐AS1 acted as a sponge of miR‐203a that also specifically targeted MMP‐2. Besides, the knockdown of lncRNA DLX6‐AS1 could suppress proliferation, migration and invasion of HCC in vitro as well as inhibiting tumour growth in vivo.^22^ In another gynaecological cancer, cervical cancer, lncRNA DLX6‐AS1 similarly functioned as an oncogene by sponging miR‐199a and promoting cell proliferation.^8^ Furthermore, Yang *et al* have shown that lncRNA DLX6‐AS1 could bind to and inhibit miR‐199a, which promoted the proliferation, migration and invasion of nasopharyngeal carcinoma cells.^23^ These various findings drew our attention to the possible mechanism between lncRNA DLX6‐AS1 and miR‐199a, which we shall explore in future studies. Much less is known about DLX6 protein in cancer. A single published report suggested that overexpression of DLX6 was associated with metastasis of the human breast cancer cell line MDA‐MB‐231.^24^ Bioinformatics analysis predicted that DLX6 protein could play important roles in embryonic development and tissue differentiation. Additionally, in the IntAct protein interaction database, DLX6 was identified to interacted with HSP 90‐beta and DNA damage‐inducible transcript 4‐like protein, which suggested an association between DLX6 and cell ROS status or DNA restoration. However, the molecular mechanism by which DLX6 protein could mediate effects of lncRNA DLX6‐AS1 to enhance tumour growth is still unknown. Hence, more investigations should be carried out to explore the function of DLX6.

Second, we found lncRNA DLX6‐AS1 could specifically bind at the promoter region of DLX6 and then recruit p300 protein to promote transcription of DLX6. Epigenetic regulation includes genome methylation and acetylation of histones or other DNA binding proteins. p300 is a histone acetyltransferase that can increase acetylation of histones and loose chromatin structure to facilitate the initiation of transcription.^25^ p300 protein shows diverse roles in various human diseases including cancers. Usually, loss of p300 protein function by mutation or knockout leads to increased occurrence of leukaemia, which suggests a tumour suppressive role of p300/CBP.^26^ On one hand, p300/CBP is indispensable to transcribe certain essential tumour suppressor genes such as p53, BRCA1 and FOXO3a. On the other hand, p300 can also serve as an enhancer of c‐Myc, c‐Myb, and AR to promote tumour growth.^27^ These dual functions suggest contradictory or opposing roles of p300/CBP in the regulation of tumorigenesis via diverse signalling pathways. For instance, p300/CBP can promote the development of prostate cancer in an AR‐dependent transcription.^28^ In this study, we proved the tumour‐activator role of p300 in an E2F1‐dependent mechanism. Interestingly, a previous report showed that lncRNA DLX6‐AS1 could relieve E2F1 by sponging miR‐197‐5p and thus promote the glioma carcinogenesis.^29^ These studies suggested that lncRNA DLX6‐AS1 could elevate gene expression through improving E2F1‐mediated transcription.

**Figure 8:**
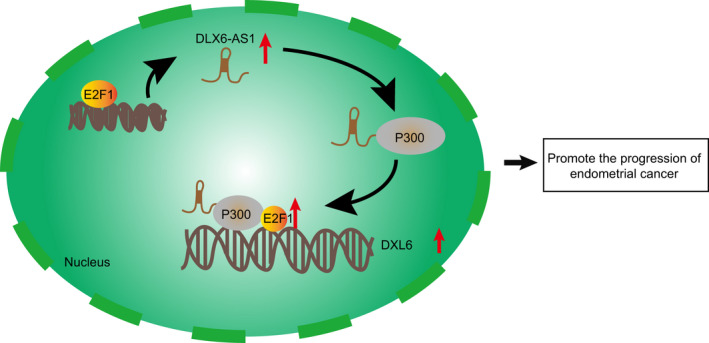
A schematic diagram depicting the molecular basis of the DLX6‐AS1/p300/E2F1/DLX6 axis in regulating the progression of endometrial cancer. To be specific, DLX6‐AS1 up‐regulates DLX6 expression by recruiting transcription factor p300/E2F1 to the DLX6 promoter region, with the end results of promoting the progression of endometrial cancer

In general, the regulation of a gene by its antisense transcript is a common mechanism in lncRNA pathways. We found that DLX6 and its antisense transcript lncRNA DLX6‐AS1 were highly expressed in endometrial cancer (Figure 8). Meanwhile, silencing lncRNA DLX6‐AS1 or DLX6 significantly suppressed tumour growth and increased cell apoptosis. Furthermore, the effects on endometrial cancer cells were dependent upon the recruitment of p300 and E2F1 via lncRNA DLX6‐AS1. Therefore, it seems that lncRNA DLX6‐AS1 and DLX6 both have oncogenic properties, which could justify efforts to develop an antagonist as an experimental treatment for endometrial cancer.

## CONFLICT OF INTEREST

The authors declare that they have no competing interests.

## AUTHOR CONTRIBUTION

**Hui Zhao:** Conceptualization (equal); Supervision (equal); Writing‐original draft (equal); Writing‐review & editing (equal). **Qian Xu:** Conceptualization (equal); Data curation (equal); Methodology (equal); Software (equal); Supervision (equal).

## Data Availability

The data that support the findings of this study are available from the corresponding author upon reasonable request.

## References

[1] Morice P , Leary A , Creutzberg C , et al. Endometrial cancer. Lancet. 2016;387(10023):1094‐1108. 26354523 10.1016/S0140-6736(15)00130-0

[2] Renehan AG , Tyson M , Egger M , et al. Body‐mass index and incidence of cancer: a systematic review and meta‐analysis of prospective observational studies. Lancet. 2008;371(9612):569‐578. 18280327 10.1016/S0140-6736(08)60269-X

[3] Reinbolt RE , Hays JL . The role of PARP inhibitors in the treatment of gynecologic malignancies. Front Oncol. 2013;3:237. 24098868 10.3389/fonc.2013.00237 PMC3787651

[4] O’Mara TA , Glubb DM , Amant F , et al. Identification of nine new susceptibility loci for endometrial cancer. Nat Commun. 2018;9(1):3166. 30093612 10.1038/s41467-018-05427-7 PMC6085317

[5] Prensner JR , Chinnaiyan AM . The emergence of lncRNAs in cancer biology. Cancer Discov. 2011;1(5):391‐407. 22096659 10.1158/2159-8290.CD-11-0209 PMC3215093

[6] Hirata H , Hinoda Y , Shahryari V , et al. Long noncoding RNA MALAT1 promotes aggressive renal cell carcinoma through Ezh2 and interacts with miR‐205. Cancer Res. 2015;75(7):1322‐1331. 25600645 10.1158/0008-5472.CAN-14-2931 PMC5884967

[7] Li T , Mo X , Fu L , et al. Molecular mechanisms of long noncoding RNAs on gastric cancer. Oncotarget. 2016;7(8):8601‐8612. 26788991 10.18632/oncotarget.6926 PMC4890990

[8] Wang X , Lin Y , Liu J . Long noncoding RNA DLX6AS1 promotes proliferation by acting as a ceRNA targeting miR199a in cervical cancer. Mol Med Rep. 2019;19(2):1248‐1255. 30535431 10.3892/mmr.2018.9729

[9] Oki S , Sone K , Oda K , et al. Oncogenic histone methyltransferase EZH2: A novel prognostic marker with therapeutic potential in endometrial cancer. Oncotarget. 2017;8(25):40402‐40411. 28418882 10.18632/oncotarget.16316 PMC5522273

[10] Li J , Li P , Zhao W , et al. Expression of long non‐coding RNA DLX6‐AS1 in lung adenocarcinoma. Cancer Cell Int. 2015;15:48. 26052251 10.1186/s12935-015-0201-5 PMC4458341

[11] Wu Q , Yi X . Down‐regulation of long noncoding RNA MALAT1 protects hippocampal neurons against excessive autophagy and apoptosis via the PI3K/Akt signaling pathway in rats with epilepsy. J Mol Neurosci. 2018;65(2):234‐245. 29858824 10.1007/s12031-018-1093-3

[12] Dunagin M , Cabili MN , Rinn J , et al. Visualization of lncRNA by single‐molecule fluorescence in situ hybridization. Methods Mol Biol. 2015;1262:3‐19. 25555572 10.1007/978-1-4939-2253-6_1

[13] Zhang E , Han L , Yin D , et al. H3K27 acetylation activated‐long non‐coding RNA CCAT1 affects cell proliferation and migration by regulating SPRY4 and HOXB13 expression in esophageal squamous cell carcinoma. Nucleic Acids Res. 2017;45(6):3086‐3101. 27956498 10.1093/nar/gkw1247 PMC5389582

[14] Xiong Y , Kuang W , Lu S , et al. Long noncoding RNA HOXB13‐AS1 regulates HOXB13 gene methylation by interacting with EZH2 in glioma. Cancer Med. 2018;7(9):4718‐4728. 30105866 10.1002/cam4.1718 PMC6144250

[15] Zhao W , Geng D , Li S , et al. LncRNA HOTAIR influences cell growth, migration, invasion, and apoptosis via the miR‐20a‐5p/HMGA2 axis in breast cancer. Cancer Med. 2018;7(3):842‐855. 29473328 10.1002/cam4.1353 PMC5852357

[16] Zhou Y‐X , Wang C , Mao L‐W , et al. Long noncoding RNA HOTAIR mediates the estrogen‐induced metastasis of endometrial cancer cells via the miR‐646/NPM1 axis. Am J Physiol Cell Physiol. 2018;314(6):C690‐C701. 29466670 10.1152/ajpcell.00222.2017

[17] Nelson JD , Denisenko O , Sova P , et al. Fast chromatin immunoprecipitation assay. Nucleic Acids Res. 2006;34(1):e2. 16397291 10.1093/nar/gnj004 PMC1325209

[18] Wang S , Ke H , Zhang H , et al. LncRNA MIR100HG promotes cell proliferation in triple‐negative breast cancer through triplex formation with p27 loci. Cell Death Dis. 2018;9(8):805. 30042378 10.1038/s41419-018-0869-2 PMC6057987

[19] Postepska‐Igielska A , Giwojna A , Gasri‐Plotnitsky L , et al. LncRNA Khps1 regulates expression of the proto‐oncogene SPHK1 via triplex‐mediated changes in chromatin structure. Mol Cell. 2015;60(4):626‐636. 26590717 10.1016/j.molcel.2015.10.001

[20] Chiu HS , Somvanshi S , Patel E , et al. Pan‐cancer analysis of lncRNA regulation supports their targeting of cancer genes in each tumor context. Cell Rep. 2018;23(1):297‐312.e212. 29617668 10.1016/j.celrep.2018.03.064 PMC5906131

[21] Zeng X , Hu Z , Ke X , et al. Long noncoding RNA DLX6‐AS1 promotes renal cell carcinoma progression via miR‐26a/PTEN axis. Cell Cycle. 2017;16(22):2212‐2219. 28881158 10.1080/15384101.2017.1361072 PMC5736339

[22] Zhang L , He X , Jin T , et al. Long non‐coding RNA DLX6‐AS1 aggravates hepatocellular carcinoma carcinogenesis by modulating miR‐203a/MMP‐2 pathway. Biomed Pharmacother. 2017;96:884‐891. 29145165 10.1016/j.biopha.2017.10.056

[23] Yang B , Jia L , Ren H , et al. LncRNA DLX6‐AS1 increases the expression of HIF‐1alpha and promotes the malignant phenotypes of nasopharyngeal carcinoma cells via targeting MiR‐199a‐5p. Mol Genet Genomic Med. 2020;8(1):e1017. 31782911 10.1002/mgg3.1017 PMC6978402

[24] Morini M , Astigiano S , Gitton Y , et al. Mutually exclusive expression of DLX2 and DLX5/6 is associated with the metastatic potential of the human breast cancer cell line MDA‐MB‐231. BMC Cancer. 2010;10:649. 21108812 10.1186/1471-2407-10-649 PMC3003273

[25] Eckner R , Ewen ME , Newsome D , et al. Molecular cloning and functional analysis of the adenovirus E1A‐associated 300‐kD protein (p300) reveals a protein with properties of a transcriptional adaptor. Genes Dev. 1994;8(8):869‐884. 7523245 10.1101/gad.8.8.869

[26] Kung AL , Rebel VI , Bronson RT , et al. Gene dose‐dependent control of hematopoiesis and hematologic tumor suppression by CBP. Genes Dev. 2000;14(3):272‐277. 10673499 PMC316359

[27] Wang F , Marshall CB , Ikura M . Transcriptional/epigenetic regulator CBP/p300 in tumorigenesis: structural and functional versatility in target recognition. Cell Mol Life Sci. 2013;70(21):3989‐4008. 23307074 10.1007/s00018-012-1254-4 PMC11113169

[28] Debes JD , Sebo TJ , Lohse CM , et al. p300 in prostate cancer proliferation and progression. Cancer Res. 2003;63(22):7638‐7640. 14633682

[29] Li X , Zhang H , Wu X . Long noncoding RNA DLX6‐AS1 accelerates the glioma carcinogenesis by competing endogenous sponging miR‐197‐5p to relieve E2F1. Gene. 2019;686:1‐7. 30366080 10.1016/j.gene.2018.10.065

